# Percussion and Auscultation

**Published:** 1842-07

**Authors:** 


### ART. XV.

1. *Die Perkussion und Auskultation.* Von Dr. JOSEPH SKODA.—*Wien*, 1841. 8vo, pp. 271.

*Percussion and Auscultation.* By Dr. JOSEPH SKODA.—*Vienna*, 1841.

2. *A Practical Treatise on Auscultation.* By MM. BARTH and ROGER. Translated, with Notes, by PATRICK NEWBIGGING, M.D.—*Edin.* 1842, Small 8vo, pp. 398.

THE several articles which we have already devoted to auscultation and percussion have detailed so fully the principal improvements and additions which have been made, up to a late period, that in recurring to these subjects at present, we have but to make a few gleanings from such of the works and essays as have fallen in our way since the date of our last article, containing anything that claims particular notice.

The treatise of Dr. Skoda makes more pretension to novelty in auscultation than we expected to meet with so soon, in a field so long explored by the active imaginations and diligent ears of the multitude who have been on the alert for every sound and fancy that might chance to occur. We wish we could compliment the author on the extent and accuracy of his acquaintance with acoustics, and on the significance of some of his new facts, as much as we feel inclined to do on the general faithfulness of his account of what has been determined by the labours of those who have preceded him. In fact it is just because this faithfulness proves him to have a good practical acquaintance with the subject, and is likely to be a passport to his book to the good opinion of the public, that we think it worth while to devote some of our space to a consideration of his errors.

The chief peculiarity of Dr. Skoda's work lies in his explanation of some of the most important phenomena observed in the voice and respiratory sounds. He has few reasons to question the received opinions of the manner in which sounds created in the larynx are made more audible than in health on that part of the surface of the chest which lies over a condensed portion of the pulmonary parenchyma, as in the case of tubercular deposit and pneumonia. He thinks that the well-known facts (1) that sound is propagated the farthest in the same medium in which it has been produced, and (2) that it is weakened by conveyance through a denser medium, in proportion to the difference of density between the media, overthrow the view which is generally entertained, viz. that bronchophony, as a sign of condensation of the pulmonary parenchyma, is a result of the better conducting power of the pulmonary parenchyma when converted into a solid; for, he argues, as the voice reaches the parenchyma through air, to wit, in the bronchial tubes, it must be conducted better to the surface of the chest by the air of the small bronchi and air-cells, than it can be by a solid interposed substance, which, according to the principles of the received law, takes up and transmits the sound in a weakened state, from the rarer medium in which it was produced. Dr. Skoda, therefore, propounds a new method of explaining the occurrence of bronchophony, and indeed of all the vocal and respiratory sounds which occur in the chest, whether in health or disease. He conceives that they depend on what is known in music by the name of *Consonance*, and is familiarly illustrated by the experiment of sounding a note on any instrument in the neighbourhood of a tuned guitar, when the string of the latter, which is capable of yielding a tone that corresponds with the note in question, emits a musical sound. This phenomenon he applies in the following way:

"The air contained in the trachea and bronchi can so far consonate with the voice as the surrounding walls have the necessary capability to reflect the sound; some, to wit the walls of the larynx, the mouth, and the nasal cavities, have such, or an analogous property. In the bronchi, whose walls consist of cartilaginous rings, the voice consonates almost as strongly as it sounds in the larynx. In a degree little less must consonance take place in the two branches into which the trachea divides. At their entrance into the parenchyma of the lungs, the bronchi, as is well known, have no more cartilaginous rings lying close to one another, but the cartilages form irregular thin plates which lie in a filamentous tissue. The plates, as the bronchi subdivide more and more, become smaller, thinner, and rarer. The fine bronchi exhibit merely thin membranous canals. Within the bronchi which run in the normal parenchyma of the lungs, the voice accordingly consonates more feebly than in the trachea, and indeed so much the weaker the more the cartilages disappear. The conditions under which the voice can consonate more strongly in the air within the bronchi in the parenchyma of the lung are—that their walls consist of cartilages, or, in case they are membranous, that they are thick, or that the surrounding tissue of the lung is devoid of air; in all these cases the walls reflect the sound more strongly than the membranous walls of a normal bronchus; it is also necessary that the air within them be in communication with the air in the larynx. When the air in a confined space is put into vibrations, primitive or derived, usually the containing walls are also put into the like vibrations, and so much the more easily, the less stiff and hard they are. . . . . The larynx vibrates at each sound, and its vibrations even allow themselves to be felt through a layer of flesh several inches thick. The walls of the bronchi running within the parenchyma of the lungs, when the air contained in them consonates with the voice, are also thrown into vibrations as well as the larynx, and these vibrations can spread through layers of flesh several inches thick, or layers of fluid, even to the walls of the chest, and we can perceive in the walls of the chest, the sound consonating in the bronchi." (p. 35.)

Then follows an account of the circumstances in which the membranous bronchi in the parenchyma of the lung may be made to reflect the sounds, transmitted from the larynx, better than in their healthy state, and thereby to produce a louder consonance. "The reflection of the sound, and the strength of the consonance are so much the greater, the firmer the parenchyma becomes." Hence the increased resonance of the voice in tubercle, pneumonia, hepatization, &c.

It may be well, before delivering our opinion of the doctrine developed in the passages we have quoted, to illustrate Dr. Skoda's capacity to give a disquisition on acoustics, by a few examples of a popular kind, with which his book furnishes us. We do this the more willingly because young and enthusiastic students or practitioners are apt to found much of their faith in novelties, on the appearance of depth and learning which their instructor exhibits in matters of which they may happen to be ignorant. In explaining his views of the general nature and laws of consonance, Dr. Skoda takes occasion to remark:

"A tuning-fork sounds much weaker when held in the air than when placed on a table or box. The table must therefore strengthen the sound, and must also make the same vibrations as the tuning-fork; it must *consonate*. The sound of a Jew's-harp is scarcely perceptible in the air, it appears much stronger when the instrument is set in motion within the mouth; the air in the cavity of the mouth must therefore increase the sound of the Jew's harp; it must *consonate* with it. .......... The air contained in the sounding box of a guitar, violin, piano, &c. consonates with the tones of the strings, whilst the free air does not increase their sound." (pp. 32-3.)

It has been well said of the Hindoos that their religion is so incorporated with their philosophy, that if the latter were exploded the former would share its fate. We augur the same of Dr. Skoda's new *auscultology;* not on the ground that he has been unhappy in his illustrations, but because they prove that he does not understand what consonance really is. The air within a guitar or violin has nothing to do, directly, with the increase of the sound of the strings, which primarily and mainly depends on the vibrations of the *wood;* and just as little has the air in the mouth to do with the augmented sound of the Jew's harp, which, as in the former case, depends chiefly and essentially on the vibrations of the bony walls of the mouth. As this little instrumeut affords a good illustration of the true principle which operates in the several examples, we may observe of it, what Dr. Skoda may easily put in practice, that the increased sound is produced only when its prongs are in contact with the teeth, and thereby communicate the vibrations of its tongue to the bones of the jaws. The pulses from the vibrating walls of the mouth, guitar, and violin being communicated to a larger body of air than that which is in contact with the steel spring, or the strings, is one main reason of the increased sound, and we are surprised that Dr. Skoda should have missed this fact, with the example of the tuning-fork and table, without any confined air, staring him in the face. The confinement of the air does indeed contribute most notably to the increase of the sound; but simply because it is instrumental in carrying from side to side the pulses of the vibrating walls, and communicating them in endless multitude and succession from one part to the other, and thereby increasing the strength of their vibrations, and thus also of its own. Now in all these examples no one but Dr. Skoda would ever dream of recognizing anything but the mere *transmission* of vibrations from one substance to another. He may choose to call it *consonance*, yet it is not the consonance hitherto known by that name; if it were, consonance would be but another name for simple transmission of sound. The wood of a guitar will give sonorous vibrations in unison with any tone of any of the strings; but the note A, B, or C of one octave will *consonate* with only the note A, B, or C of another. This new theory of the phenomena of auscultation, therefore, is founded on a misconception of the laws and phenomena of acoustics, and the *consonance* of Dr. Skoda is but the ordinary *communication of sound* of other authors.

It is clear that if bronchophony in a piece of diseased pulmonary parenchyma be produced by the same means as the full sound of a guitar-string, it must be mainly owing to vibrations in the walls of a bronchus, which correspond to the wood of the guitar; but this is at variance with Dr. Skoda's theory and with fact. Again, if it be produced, independently of vibrations of the bronchi, in the column of air which is continuous with the air in the larynx, where the sound is created, it cannot be from consonance, else one end of a string, piece of wood, &c. may be said to consonate with the other, and there can be no such thing as simple transmission of sound. All that he has taken the trouble to say about the loss of strength in sounds transmitted from one medium to another, owing to the reflection that takes place at their surfaces of contact, and which he illustrates by referring to sounds produced in water and in wood, which are well known to lose a great deal in the transmission, has little to do with the circumstances in which sounds are propagated through the chest. These sounds are propagated in confined air, the vibrations of which are constantly striking and restriking against the containing walls, and are not driven off into free space, as a sound produced in the open air is from the surface of water or wood. That solids surrounding confined air in a state of sonorous vibration take on the vibration in much force is well exemplified by what occurs in the nasal cavities. Let Dr. Skoda put his hand on the top of his head, and direct the nasal sound of the letter *n* into these cavities, and he will become sensible of a force of vibration that would penetrate a Bœotian skull. It is precisely on the same principle that bronchophony is sensible to the ear over a piece of hepatized lung. The many reflections and multiplied vibrations of the contained air set the bronchial walls into vibratory motion, and then the more solid the tissue on the outside of them the better will the sound be conducted. A worse conducting medium than the healthy parenchyma could scarcely be invented. It is true that the uninterruptedness of the column of air from the larynx to the peripheral surface of the lungs must conduce to the facility and force with which sounds created in the larynx are transmitted to the walls of the chest; yet but a small portion of that sound is so conducted, since the vibrations conveyed by all the tubes, with the exception of the minute twigs, which terminate in the lobules that compose the exterior surface of the lung, must be damped and interrupted in their progress to the surface by the innumerable alternations of membrane and air in the deeper lobules. The continuous consolidation of such a tissue by a substance which would render the medium of the transmission of sound from deep-seated bronchi of some size, to the surface, of a greater and at the same time more uniform density, cannot but contribute to augment the strength of the sounds heard at the surface, and we are constrained to abide by the opinion of Laennec, and stethoscopists in general, that such is the true cause of bronchophony and bronchial respiration. As to the objection of Dr. Skoda to the better-conducting power of solids, implied in his reference to the general preference of hollow stethoscopes, we have to observe only that solid ones certainly answer very well; and if there really be a good reason for the preference, which is doubtful, we think this may be explained on other principles than those of Dr. Skoda.

Among the valuable practical observations of Dr. Skoda on the phenomena of the voice in hepatization, and the same applies to tuberculation, of the lung, one which has been very generally overlooked, even by writers on auscultation, is the effect of mucus in the bronchial tubes in suspending bronchophony. No one can have studied the signs of condensation very often without having been perplexed by the occasional absence of bronchophony and bronchial respiration, while the other signs and the general symptoms indicated advanced pneumonia or phthisis. Coughing and expectoration usually restore the suspended sounds, and betray the cause of their temporary suspension. In consumption, expectoration is not always so complete as to clear the tubes sufficiently, and then in not a few instances, in which impaired percussion-sound and small mucous rattles are the only direct proofs of the condensed state of the parenchyma. We have noticed also this presence of mucus in the bronchial tubes of tubercular condensation prevent the communication of the vocal thrill to the surface of the chest, over the condensed part, or greatly to lessen it. The tendency of condensation is to increase the power of the parenchyma to conduct the voice to the surface, and the vocal thrill, perceptible by the fingers, consists of the identical vibrations which constitute the sound. The force of the voice itself cannot be much augmented by the condensation without the usual thrill being also increased; and we conceive the difference of opinion which has existed respecting the state of the thrill in condensation to be owing to inattention to the contents of the bronchi. We have known the thrill on the side on which tubercular condensation affected the upper part of the lung to have been so strong as to be felt with inconsiderable force on the extreme point of the shoulder.

We cannot follow Dr. Skoda through the details of his work; but before quitting that part of it which is devoted to diseases of the respiratory organs, we must protest against the sentiment that liquid effusion in the pleura is usually a cause of bronchophony, and that several pints of it frequently coexist with that phenomenon. MM. Barth and Roger express themselves on this point much more accurately than Dr. Skoda, whose observations are too general by far:

"Some authors," say they, "consider the existence of bronchophony in pleurisy as very common, and explain it by the compression of the pulmonary parenchyma and the smaller branches of the bronchi, whereby the vocal vibrations are concentrated in the large bronchi. For ourselves, however, we cannot admit that proposition without restrictions. We do not deny the existence of *bronchial resonance* in pleuritic effusions, but we hold that it is distinguished by peculiar characters from true bronchophony, such, for instance, as is observed in pneumonia.......... Bronchophony does not properly belong to pleuritic effusion: this latter state is more clearly characterized by another vocal resonance, more remarkable for its tone than for its loudness, viz. ægophony." (Newbigging's Translation, pp. 125-6.)

These authors attach, perhaps, too much importance to this last sign as a result of pleuritic effusion, though they admit that it may depend on effusion of coagulable lymph alone, yet we conceive them to be much nearer the truth than Dr. Skoda, when he says that he has noticed ægophony as often without fluid in the pleura as with it, in pneumonia also, and in tubercular infiltration of the lungs, with and without excavation (p. 58); and when he states that three or four ounces of fluid cannot of themselves give rise to ægophony, and that when it is not present naturally, as he maintains it to be in certain children and females, it cannot be heard when fluid is in the pleura, except when the quantity of fluid is so great, that a piece of lung, containing a considerable bronchus, is compressed and destitute of air, (p. 62.) In short, Dr. Skoda makes it merely an accidental variety of bronchophony which may be audible in any of the diseases which can produce the conditions necessary for his consonance. We believe that there are more circumstances than fluid or coagulable lymph in the pleura, capable of giving rise to ægophony; but we doubt much the accuracy of Dr. Skoda's statements, to the sweeping extent we have noticed, in respect to true

ægophony. We have heard something like it produced by the acute female voice in a healthy state of the lung, and something like it, also, in œdema of the lungs, but certainly never the true tremulous bleating sounds, where there was much fluid, or in pneumonia. A strong objection to Dr. Skoda's view of its production by the compression of a part of the lung containing a considerable bronchus, by a large effusion, lies in the fact that when ægophony is audible after a good deal of fluid has collected, it is not at the point where the compression is nearly the greatest but at the upper margin of the sphere of impaired percussion-sound, where the stratum of fluid is thin, and the compression consequently slight.

We pass on to Dr. Skoda's account of auscultation of diseases of the heart, of which we have a few words to say. The chief novelties which Dr. Skoda announces in respect to the phenomena of diseases of the heart, relate to the intensity of the second sound, unchanged into a murmur, as heard on parts of the chest corresponding to the aorta, and to the pulmonary artery. It had been observed by former writers, and is particularly stated by Dr. Hope, that in certain circumstances the second sounds, in the aorta and in the pulmonary artery, underwent an increase. He mentions this in general terms, as the result of hypertrophy of the ventricles. Dr. Skoda is more particular, and affirms that the augmentations of the second sound in the pulmonary artery can be distinguished from that in the aorta, by being heard best to the left of the sternum, while the latter is audible with most intensity on the bone, and even to the right side of it. We are satisfied of the accuracy of these statements, which we have often tested when such diseases were present. in the one or in the other ventricle, as were calculated to produce a difference in the strength of the second sounds of the two vessels. The level of the third cartilage to the left of the sternum, or of the interval between the second and third cartilages, is the proper point for ascertaining the intensity of the second sound in the pulmonary artery; and the left side of the sternum, or its middle on the same level, or even further at the bone, is the proper place for studying the intensity of the second sound in the aorta. The more the arteries are distended by the force of waves of blood propelled suddenly into them, the more forcibly do they react, and the quicker and greater is the tension of the semilunar valves. So far, that is, to the extent of determining the degree of power with which the ventricles, or either of them, contract, the intensity of the second sound is a valuable criterion, *provided* there be (by percussion of the region of the heart and otherwise) evidence that the bulk of the heart is increased. This limitation is essential to an accurate diagnosis, for, even when the left ventricle is of its natural size, an increase in the strength of the second sound, of the aorta often, if not always, occurs as a consequence of widening of the ascending part of the vessel, an occurrence overlooked by Dr. Skoda.

An increase in the strength of the second sound in the pulmonary artery is dwelt upon by the author as of great consequence in the diagnosis, not merely of hypertrophy of the right ventricle, but of the origin of valvular murmurs. His fundamental doctrine on this particular is, that incompetency of the left auriculo-ventricular opening allows of so much regurgitation into the auricle, and therefore causes so great a hinderance to the transmission of blood through the pulmonary veins and pulmonary artery, that a distended condition of the latter is maintained and a more forcible reaction of the blood within it produced, in consequence, against the valves, when the systole of the ventricle is over. Whether this is the true explanation of the increase of sound, and not rather that it is owing to the hypertrophy of the right ventricle which so constantly attends incompetency of the left auriculo-ventricular opening, we shall not pause to enquire, but proceed to examine the significance of the sign in pointing out valvular disease. When, says Dr. Skoda, there is a murmur in the left ventricle during the systole, it may be concluded to depend on incompetency of the mitral valve, if the second sound in the pulmonary artery be increased; and if it be not, then the murmur signifies roughness of the aortic valves, or of the inner membrane of the ventricle, near the mouth of the aorta. (p. 184.) Now, if we admit with Dr. Graves (Dub. Journal, May, 1842), that a murmur created in the aorta may be heard chiefly in the region of the left ventricle, and allow also that this may coexist with hypertrophy of the right ventricle, a circumstance which may certainly occur, of what value does the increase of the second sound in the pulmonary artery become? It then will depend simply on the state of the right ventricle, and would undoubtedly mislead if Dr. Skoda's rule were trusted to. The increase of the second sound in the pulmonary artery cannot therefore be considered as more than a curious phenomenon, certainly not as a trustworthy guide to the diagnosis of the diseased orifice from which a murmur proceeds.

At p. 185, Dr. Skoda observes that when there is neither any natural sound (ton) nor murmur audible during the contraction of the left ventricle, it is of especial consequence to take notice of the second sound in the pulmonary artery, and that a natural sound or murmur occurs during the diastole of the ventricle, for this end, that if the second sound in the pulmonary artery be not increased, and the natural second sound in the left ventricle be audible, it may be concluded, according to his experience, that the mitral valve is competent; and, on the contrary, if the second sound in the pulmonary artery be increased, and a murmur occur instead of the natural second sound in the left ventricle, that the mitral valve is not competent. To both of these propositions there are such objections as prove them to be no better than ingenious refinements, the offspring of closet-meditation. In respect to the first, an assertion of his own at p. 185 shows the weakness of the conclusion. He there states that sometimes the mitral valve is incompetent, while yet no murmur attends the regurgitation into the auricle to prove its incompetency, and we have already seen that mere hypertrophy of the right ventricle may produce an increase in the second sound of the pulmonary artery. There will, then, be no murmur anywhere; the second sound may be present in the left ventricle, and increased in the pulmonary artery, yet incompetency of the mitral valve may exist. If the second proposition be not a misstatement, owing to a typographical error, it must be laid, with the other, to the account of the author, as a semeiological mistake. A murmur instead of the second sound in the left ventricle, if it ever proceed from the auriculo-ventricular opening, cannot be caused by *incompetency* of the mitral valve, though possibly it may by narrowing or roughening of the orifice, which is not necessarily connected with insufficiency of the valve. The increase of the second sound, in this proposition, may depend, as in the other, on hypertrophy of the right ventricle.

There is much more of this pseudo-generalization respecting the sounds of the heart; but we suspect that by this time our readers are satisfied with our notice of Dr. Skoda.

We gave a brief notice of the work of MM. Barth and Roger in our last Number. It is, as we then stated, a sensible and accurate monograph on the subjects of which it treats. Dr. Newbigging's translation, as we then stated also, is remarkable for its faithfulness, and cannot but prove acceptable and highly useful to the English student. Indeed, the faithfulness of the translation is its only fault; and a very intelligible one it is, in the case of an over-anxious and modest translator, who has more respect for his original than confidence in himself. In his next edition, Dr. Newbigging will doubtless be less timid, and translate more freely, as his knowledge of the subject entitles him to do. He might add much to the value of the work by notes on the diagnosis of aneurism more especially, for which he will find ample materials in the journals, and by a chapter on percussion.

Among the more recent contributions to our journals on the subject of auscultation, we may name that of Dr. Bennett, in the Edinb. Monthly Journal, for February, 1842, as one of most pretension, and, to say truth, also one of the best. The readers of Piorry, however, will find in it much less of novelty than its general tone would lead to infer. The most important novelty in it is the account of a new hammer by Dr. Winterich of Wurtzburg, for the practice of percussion. In common with most of our brethren in this country, we have been accustomed for many years to employ the fingers of the right hand as a percussor, and one of the fingers of the left as a pleximeter, and we still think this method sufficient in all ordinary cases, as it is decidedly the most convenient to the physician and least formidable to the patient. We have, however, often used other pleximeters of ivory, wood, leather, India-rubber, &c., and we think them preferable to the finger in certain cases and under certain circumstances. We have also used, occasionally, one or other of the numerous hammers that have been proposed, by different auscultators, from time to time. But these last we have always shortly relinquished, from conviction that they were not merely unnecessary but disadvantageous. To say nothing of the trouble of carrying a large additional instrument, and the effect of so formidable an apparatus on the mind of timid patients,—minor evils we admit, and which of course must give way to great and positive advantages, if such are found to attend its use,—we have been accustomed to regard the loss of the tactual impression conveyed to the percussing finger involved in the use of the hammer, as a fatal objection to its employment. We must confess, however, that our experience of Dr. Burne's, or any other of the hammers heretofore in use, has not been, for the reasons mentioned, very great, and that our employment of Dr. Winterich's is still only of very recent date; we therefore do not feel ourselves justified either in condemning the use of this mode of percussion generally, or in denying to the hammer of Dr. Winterich the great superiority which Dr. Bennett claims for it. We shall not fail to give this last a fair trial; and in the meantime we recommend our readers to consult Dr. Bennett's account of it and of its great advantages. It is more especially recommended to the clinical physician who has to enable others to hear as well as himself.

By the way, Dr. Bennett is unhappy in one of his designations for the characters of the sounds yielded by percussion; we allude to the word *humoral*. If he intend this to correspond to the *bruit humorique* of Piorry he has mistaken the nature of the latter, which is better indicated by the synonyme *hydro-pneumatique*, occurring as it does only when air and liquid are in contact. If, on the other hand, he intend it to signify a sound peculiar to liquid alone, we suspect that it is no improvement on the general term dulness, the dull sound of accumulated fluid possessing no difference from a thoroughly-dull sound depending on any other cause.

The student must not be scared by the apparent intricacy of mediate percussion as detailed by Dr. Bennett, for we can assure him that not a few of the directions are over-nice, and some of an impracticable exactness. Of the latter sort is the following reference to two or three ounces of liquid effusion in the cavity of the pleura: "It is readily distinguished," he says, "posteriorly, from the dulness of the liver on the right side," independently, as appears from the context, of change in the posture of the patient, and owing to the supposed humoral sound and diminished sense of resistance. Abundant observation convinces us that so near the liver there is not such a diminished sense of resistance on percussion as can be of value in forming the diagnosis.

---

